# Future Therapeutic Strategies for Freezing of Gait in Parkinson’s Disease

**DOI:** 10.3389/fnhum.2021.741918

**Published:** 2021-11-02

**Authors:** Cathy K. Cui, Simon J. G. Lewis

**Affiliations:** ForeFront Parkinson’s Disease Research Clinic, Brain and Mind Centre, School of Medical Sciences, The University of Sydney, Camperdown, NSW, Australia

**Keywords:** gait disorders, dopamine agents, deep brain stimulation, non-invasive stimulation, physical therapy, repurposing, problem solving, humans

## Abstract

Freezing of gait (FOG) is a common and challenging clinical symptom in Parkinson’s disease. In this review, we summarise the recent insights into freezing of gait and highlight the strategies that should be considered to improve future treatment. There is a need to develop individualised and on-demand therapies, through improved detection and wearable technologies. Whilst there already exist a number of pharmacological (e.g., dopaminergic and beyond dopamine), non-pharmacological (physiotherapy and cueing, cognitive training, and non-invasive brain stimulation) and surgical approaches to freezing (i.e., dual-site deep brain stimulation, closed-loop programming), an integrated collaborative approach to future research in this complex area will be necessary to systematically investigate new therapeutic avenues. A review of the literature suggests standardising how gait freezing is measured, enriching patient cohorts for preventative studies, and harnessing the power of existing data, could help lead to more effective treatments for freezing of gait and offer relief to many patients.

## Introduction

**FIGURE 1:**
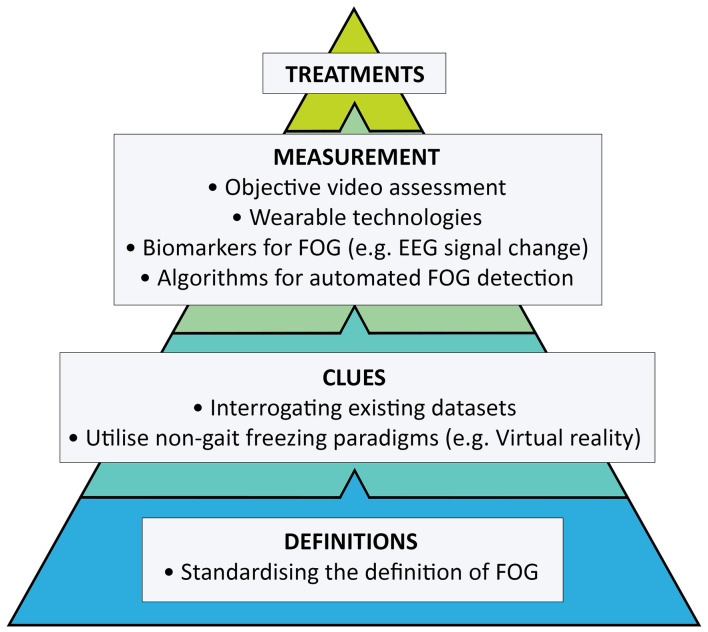
Key steps toward developing effective therapies for freezing of gait.

Freezing of gait (FOG) is a disabling symptom that affects more than half of all advanced Parkinson’s disease (PD) patients (Giladi et al., 2001b; Forsaa et al., 2015; Zhang et al., 2021). It profoundly reduces quality of life (Perez-Lloret et al., 2014; Walton et al., 2015b), leading to falls (Okuma et al., 2018; Lieberman et al., 2019) and a loss of independence. Patients who develop gait freezing fare poorly: falls related to gait freezing occur during walking, rather than standing, resulting in more severe injuries and increased hospitalisation (Lieberman et al., 2019). Gait freezing is also associated with a higher burden of non-motor symptoms (Choi et al., 2019) and femoral neck osteoporosis (Choi et al., 2021), independent of disease duration and stage of disease, which has implications for the broader treatment of such patients. Our understanding about the pathophysiology underpinning FOG is improving to appreciate its episodic features, heterogeneous phenotypes (Schaafsma et al., 2003) and the variety of modulators that can both trigger and relieve attacks (Ehgoetz Martens et al., 2018b). However, the symptom remains a treatment challenge. Whilst several established approaches, such as physiotherapy and optimising dopaminergic therapy, have long formed the cornerstones of management, FOG appears more difficult to treat compared to other Parkinsonian symptoms. Specific triggers of FOG differ between individuals, and successful treatment is likely to require the identification and targeting of these features at the level of the individual. However, intervention studies tend not to stratify participants by phenotype (Ehgoetz Martens et al., 2018b). Even more foundational, the first hurdle in identifying better treatments is of accurately and objectively measuring FOG itself. This review will highlight where future strategies need to be directed in our pursuit of more effective therapies (Figure 1).

## Pathophysiology of Gait Freezing

Unfortunately, FOG has a complex pathophysiology that is only somewhat understood. Critical anatomical areas involved in locomotion are the pontomedullary reticular formation (PMRF), mesencephalic locomotor region (MLR) including the pedunculopontine nucleus (PPN), basal ganglia and frontal cortical regions (Nutt et al., 2011). These supraspinal structures act on central pattern generators in spinal segments, which are involved in basic rhythmical stepping (Guertin, 2009). Transient disruption of this locomotor circuitry is thought to be responsible for FOG: Nieuwboer and Giladi (2013) have summarised four current models in the literature seeking to explain its episodic nature. Firstly, the “threshold” model suggests FOG manifests when multiple motor gait abnormalities accumulate to a critical threshold of instability, leading to gait breakdown (Plotnik et al., 2005). Secondly, the “interference” model proposes FOG arises from cross-talk between parallel cognitive and limbic circuits passing through the basal ganglia inducing temporary inhibition of the PPN (Lewis and Barker, 2009). The third “cognitive” model is conceptualised as a conflict-resolution deficit, related to executive dysfunction, where freezers are unable to compensate in complex situations for deficits in automaticity by switching to increased cognitive control, resulting in gait breakdown (D’Ostilio and Garraux, 2012). Lastly, the “decoupling” model refers to a discrepancy between perceived intention to move, and failure of a pre-planned motor program that then propagates motor arrest (Jacobs et al., 2009). Each model is likely to contribute to FOG, with various degrees of interplay in an individual patient, and resulting in its heterogeneity (Nieuwboer and Giladi, 2013). Situational factors such as anxiety and dual-tasking (Hackney and Earhart, 2010; Ehgoetz Martens et al., 2018b) may trigger FOG through a combination of models. In the background, the likelihood of a FOG episode occurring will increase with progression of disease, as cognitive and motor reserve is eroded and the response to levodopa becomes more variable (Giladi et al., 2001b; Nonnekes et al., 2020). Despite the complexity of these mechanisms, models such as these provide a theoretical framework for current and future treatment strategies such as reducing neural overload or improving motor gait parameters.

## How Would We Confirm an Effective Treatment?

One of the first considerations when thinking about the development of an effective therapy for FOG, is just how to go about measuring the symptom itself. The current consensus statement defines FOG as the “brief, episodic absence or marked reduction of forward progression of the feet despite the intention to walk” (Nutt et al., 2011). This definition followed on from an earlier proposal that FOG represents “an episodic inability (lasting seconds) to generate effective stepping” (Giladi and Nieuwboer, 2008). However, these definitions whilst helpful in the clinic do not reflect the complexity of FOG (Nutt et al., 2011) and do little to establish objective criteria that can be generalised for objective trial work. For example, a variety of FOG phenotypes (Schaafsma et al., 2003) have been described, along with typical phenomena including start hesitation and target freezing (Giladi et al., 1992). There are three phenotypes based on leg movement: (i) shuffling with small steps, (ii) trembling in place, and (iii) complete akinesia (Schaafsma et al., 2003), with complete akinesia occurring much less frequently than the others (Schaafsma et al., 2003). Whilst most FOG is “off” FOG, which is relieved by dopaminergic medication, less common types include “pseudo-on” FOG which is seen during a seemingly “on” state but improves with additional dopamine, and true “on” FOG, which appears induced by dopaminergic stimulation (Espay et al., 2012). We would like to highlight that there is very little evidence to explain the pathophysiology underpinning these sub-types and we would like to avoid being too speculative. Thus, we have focused on the pragmatic basis for treating the broader issue. It is unclear as to whether these different manifestations of FOG share the same underlying mechanisms (Ehgoetz Martens et al., 2018b; Mancini et al., 2019), and therefore, it is difficult to know if they are comparable for scoring purposes in an intervention study. Furthermore, most patients exhibit mixed patterns of FOG (Giladi et al., 1992) and it is not clear if the most appropriate measure would be to compare the impact of any novel treatment on the total amount of time spent freezing or if each component of FOG (e.g., periods of start hesitation, festination) should be compared separately.

Confirming any effective treatment would also require an accurate and objective measurement of FOG (Mancini et al., 2019; Table 1). This is surprisingly difficult as FOG is most commonly experienced unpredictably at home where gait is more automatic or natural. In the clinic, gait becomes more goal directed and it can become difficult to trigger episodes (Mancini et al., 2019). Two questionnaires for the assessment of FOG have previously been developed, namely the Freezing of Gait Questionnaire (FOG-Q; Giladi et al., 2000) and the New Freezing of Gait Questionnaire (NFOG-Q; Nieuwboer et al., 2009). Whilst these were both validated in sizeable cohorts against subjective carer and clinician ratings, there was no gold standard measure or definition of FOG at the time the instruments were constructed (Nieuwboer et al., 2009). Indeed, subsequent work has demonstrated that self-perceived ratings of FOG severity using the FOG-Q and NFOG-Q do not correlate well with the actual number or duration of objective freezing episodes when scored from video recordings of Timed Up and Go (TUG) walking tasks (Shine et al., 2012). Furthermore, it is only recently that the authors of the NFOG-Q examined its test-retest reliability, as well as its ability to detect minimal change. This work found that the NFOG-Q is not sufficiently reliable or responsive to detect small effect sizes (Hulzinga et al., 2020).

**TABLE 1:** Assessment methods in use for FOG measurement, and their advantages and disadvantages.

Assessment method	Advantages	Disadvantages
**Self-reported FOG** FOG-Q (Giladi et al., 2000) NFOG-Q (Nieuwboer et al., 2009)	■ Records FOG over different environments including at home ■ Assesses impact on quality of life ■ Ease and speed of administration	■ Relying on patient or carer recognition of FOG, though the NFOG-Q comes with accompanying video demonstrating FOG, making it easier to improve its recognition ■ May not detect small effect sizes (Hulzinga et al., 2020) ■ Scores do not correlate with frequency or duration of observed freezing (Shine et al., 2012)
**Gait parameters** Timed up and Go (Podsiadlo and Richardson, 1991) Walking biometrics (cadence, step length, step variability)	■ Measures functional mobility ■ Simple to perform	■ Not specific to FOG ■ Step biometrics require specialised equipment (gait pressure-mat)
**FOG-provoking tasks** Stepping in place (Nantel et al., 2011) Walking course (Ziegler et al., 2010) Virtual reality walking course (Shine et al., 2013a)	■ Set walking course or task standardises FOG triggers across subjects ■ FOG provoking tasks (e.g., dual tasking, turning, doorway walking, approaching target) can be incorporated to more reliably elicit FOG in laboratory settings ■ Virtual reality walking allows manipulation of the walking environment (e.g., increase threat and anxiety) to assess their impacts on FOG (Ehgoetz Martens et al., 2015)	■ Could be less sensitive to FOG as gait becomes more goal directed and less automatic ■ Subjects requiring gait aids or those likely to fall may not be safe to complete the tasks
**Visual scoring of FOG** Video (Morris et al., 2012) Live rater	■ Facilitates quantification of FOG (e.g., FOG duration, number of episodes, % time frozen) ■ Video data is easily shared between multiple raters ■ Ability to adjust play-back speed and replay video to identify short FOG	■ Less sensitive to FOG as gait becomes more goal directed ■ Time-intensive processing by human raters ■ Variability between clinicians’ ratings across centres, more so in the live setting ■ Algorithms for automatic video processing not yet at high accuracy
**Instrument-based freezing indices** Accelerometer [Freezing Ratio (Mancini et al., 2017)] Pressure mat Electromyography Smart phone Combination	■ Allows for faster processing speed if using automated algorithm	■ Requires specialised and often bulky equipment, again limiting assessment in the home ■ Body-worn sensors may interfere with normal gait
**Home-based wearable devices**	■ Captures more automatic gait in the everyday environment ■ Allows for long-term monitoring ■ Could deliver a therapeutic intervention (e.g., cue)	■ Artefact and interference ■ Devices need to operate at a patient or carer level of expertise, which may limit complex or bulky set-ups

In an effort to generate more objective measures, some researchers have developed standardised FOG assessments, such as the FOG Score (Ziegler et al., 2010), freezing indices based on accelerometer data (Moore et al., 2008; Mancini et al., 2012), and Stepping in Place on a pressure mat (Nantel et al., 2011). The FOG score is a clinical rating tool that scores freezing episodes as a subject completes four tasks aimed to elicit freezing (gait initiation, turning clockwise, turning counter-clockwise, and passing through a doorway), with and without two types of dual task (Ziegler et al., 2010). This method can objectively measure FOG severity, is sensitive to On and Off-medication states, and correlates well with patient self-evaluation of FOG. However, the duration of FOG episodes is not considered and the FOG Score assumes that akinetic freezing is of greater severity than the festination phenotype (Ziegler et al., 2010).

Instruments such as accelerometers (Moore et al., 2008; Mancini et al., 2012, 2017), force plates under the feet (Nantel et al., 2011), and lower limb surface electromyography (Nieuwboer et al., 2004) have all been used in the gait laboratory setting to quantify freezing along with a range of algorithms to produce an automated FOG detection mechanism. Previously, researchers have shown that body-worn inertial sensors can record a Freezing Ratio during a 2-min turning in place protocol that correlated well with clinical ratings of FOG (Mancini et al., 2017). However, these instrumented algorithms have not been widely validated for FOG assessment outside of their specific research purpose.

Visually scoring FOG from video by independent raters is still currently recognised as the gold-standard for assessing FOG severity in PD (Morris et al., 2012; Shine et al., 2012; Walton et al., 2018). This approach can be used to calculate the percentage of time spent frozen during a TUG task and has demonstrated excellent inter-rater correlations (Morris et al., 2012; Walton et al., 2018). However, this approach is time consuming to score and does not reflect what might be occurring outside of the clinic. In future, automated video scoring (Hu et al., 2020) could make this approach more viable at scale for comparing between assessment centres in the setting of a clinical trial. Obviously, there is a need for reliable, portable home based sensors or wearable technologies (Silva de Lima et al., 2017) that could identify even brief episodes of FOG during everyday activities and this is becoming a more focused area of FOG research (Marcante et al., 2020; Mancini et al., 2021).

## What Should We Focus on Treating?

Not surprisingly, most current research trials are focused on symptomatic therapies for patients with established FOG (see below), rather than exploring approaches to delay or prevent the onset of freezing. However, some data does exist about these “at risk” groups (Gao et al., 2020) and identifying those patients who will go on to develop FOG is of great interest given that they may benefit from specific intervention approaches, such as physiotherapy (Cosentino et al., 2020) or cognitive behavioural therapy (Moonen et al., 2021).

There are only a limited number of longitudinal studies that have followed patients without freezing to explore those characteristics that are associated with the future emergence of FOG, and whilst highlighting some of the potential risk factors for developing FOG, more integrated studies looking across further potential variables are probably required to understand the pathophysiological mechanisms by which they might be operating (Giladi et al., 2001a; Forsaa et al., 2015; Zhang et al., 2016; Ehgoetz Martens et al., 2018a; Kim et al., 2018; Kim R. et al., 2019; Gallea et al., 2021). These studies have identified that whilst patients with FOG have higher depression scores earlier in their disease course (Giladi et al., 2001a), the presence of anxiety may be more predictive of FOG onset within the next 12 months (Ehgoetz Martens et al., 2018a). More generally, a higher burden of neuropsychiatric symptoms predicted earlier onset of freezing of gait in a 2-year prospective study of 329 drug-naïve patients with PD, after adjusting for age of onset, disease duration, Unified PD Rating Scale (UPDRS) motor score, and dopamine transporter (DAT) activity (Jeong et al., 2021). Other clinical factors such as non-tremor predominance, early gait disturbance, cognitive impairment, left-sided disease onset and higher daily levodopa have also been associated with the development of FOG (Giladi et al., 2001a; Forsaa et al., 2015; Zhang et al., 2016; Kim et al., 2018; Lichter et al., 2021).

Other novel approaches for identifying those patients at risk of developing FOG are also being described. One recent study found that compared to a non-freezer group, patients who developed freezing within 5 years demonstrated increased baseline anti-saccade latencies (>300 ms), whilst having equivalent motor and cognitive deficits (Gallea et al., 2021). Indeed, this parameter alone was also strongly predictive for the presence of FOG and correctly classified 88% of non-freezers and 76% of eventual freezers (Gallea et al., 2021), which is broadly consistent with earlier work showing anti-saccade errors in PD patients with FOG (Walton et al., 2015a). Increased anti-saccade latencies were also correlated with decreased connectivity in the mesencephalic locomotor region-supplementary motor area (MRL-SMA) network, one of the networks involved in gait control, and a compensatory increase in other networks years before onset of freezing, which might provide a potential neurobiological explanation for these associations (Walton et al., 2015a).

It is also possible that biomarkers might prove useful in identifying those non-freezers at greatest risk of transitioning to FOG. Severe reduction in DAT activity in the caudate and putamen is associated with significantly higher incidence of FOG (Kim et al., 2018). Previous neuroimaging studies have identified the potential contribution of cholinergic deficits to FOG (Mancini et al., 2019), and amongst CSF biomarkers, low β-amyloid 1–42 has been associated with the future development of FOG in early stage PD patients (Kim R. et al., 2019). Obviously, it is not known whether this finding represents the role of concomitant Alzheimer-type pathology and it is well known that FOG is associated with cognitive decline (Irwin et al., 2012). Furthermore, combining β-amyloid 1–42 levels in a model integrating caudate DaTscan uptake and the postural instability gait difficulty (PIGD) motor phenotype score performed even better in identifying future freezers (Kim R. et al., 2019).

Thus, mechanisms already exist for enriching patients at risk of developing FOG who might be suitable for intervention studies. Such enriched cohorts would not only be a target group for early treatments, but may also reduce costs of recruitment and follow-up if accelerated FOG development is accounted for in a trial design.

## Pharmacological Approaches

It is well known that FOG occurs more frequently in the Off-state (Schaafsma et al., 2003) and thus, the first line treatment for Off-freezing is manipulating dopaminergic therapies to reduce Off time (Fietzek et al., 2013; Nonnekes et al., 2015). Studies evaluating that adjunct use of the monoamine oxidase B (MAO-B) inhibitors selegiline (Iijima et al., 2017) and rasagiline (Rascol et al., 2005; Cibulcik et al., 2016; Rahimi et al., 2016) have reported reductions in FOG, presumably through this mechanism. There is no available data yet to confirm whether the newest agent in this class, namely safinamide, may also be helpful in this regard. Freezing of gait was not an endpoint in the major randomised controlled trial of safinamide for wearing Off symptoms (Borgohain et al., 2014), but interestingly FOG-Q scores did not improve in a smaller recent uncontrolled study of 50 patients (Garcia et al., 2021).

The phenomenon of On-freezing is less common and much more difficult to manage as its relationship to dopamine levels is not fully understood (Espay et al., 2012; Cossu et al., 2015; Morales-Briceno et al., 2020). A recent proposal has suggested that levodopa may trigger FOG, hypothesising that maladaptive plasticity might in fact be induced by levodopa, which disproportionally increases the mismatch between motor and non-motor (cognitive and limbic) loops (Nonnekes et al., 2020). Obviously, the need by most patients for levodopa may limit meaningful investigation of this phenomenon but one approach might be through a large prospective delayed start design to see whether the earlier use of levodopa may drive the development of FOG. However, it should be highlighted that maladaptive plasticity may only occur with severe levels of striatal dopamine depletion and much of the literature supporting the paradox was recorded in the pre-levodopa era. Interestingly, a recent case series of five PD patients treated with 24-h levodopa carbidopa intestinal gel (LCIG) infusion, has reported a reduction in levodopa-unresponsive freezing and falls, when compared to conventional 16-h LCIG (Chang et al., 2015). The mechanisms underpinning such a finding are unclear, although improvements in sleep were proposed by the authors.

Though degeneration of dopaminergic neurons is the pathological hallmark of PD, non-dopaminergic neurons are also lost in the disease (Kalia et al., 2013). Cholinergic deficits related to cholinergic neuronal loss in the pedunculopontine nucleus (PPN) and nucleus basalis of Meynert (Karachi et al., 2010; Yarnall et al., 2011) have been reported as contributing to gait (Rochester et al., 2012) and attentional disturbance (Bohnen et al., 2006). Furthermore, antimuscarinic use has been found to be more frequent in the FOG group compared to non-FOG, in a cross-sectional study of 672 PD patients (Perez-Lloret et al., 2014). More recently, a phase 2 placebo-controlled trial of 130 PD patients found that the acetylcholinesterase inhibitor rivastigmine, improved step time variability, falls per month, gait speed whilst dual-tasking and freezing during the last month of a 32-week trial (Henderson et al., 2016). However, FOG was not a primary endpoint of this trial and a larger phase 3 trial aiming to recruit 600 patients is currently underway (ClinicalTrials.gov, 2021a).

Drugs that enhance noradrenergic transmission have also been investigated for FOG, given its possible association with noradrenergic neuron loss in the locus coeruleus (Rommelfanger and Weinshenker, 2007; Ono et al., 2016). However, current trials have been disappointing including two small, randomised studies of Atomoxetine, a selective noradrenaline reuptake inhibitor, which failed to improve dopamine-resistant FOG (Jankovic, 2009; Revuelta et al., 2015). Limited open-label data for droxidopa (L-threo-3,4-dihydroxyphenylserine), a noradrenaline precursor licensed for use for orthostatic hypotension, has suggested that it may be useful in combination with entacapone for treating dopamine-resistant FOG (Fukada et al., 2013). However, it is difficult to know how much of this response related specifically to stimulation of the noradrenergic pathways. Similarly, methylphenidate is a drug that increases both synaptic noradrenaline, as well as dopamine levels. Previous trials of methylphenidate have reported mixed results where FOG-Q scores were improved in patients with advanced disease who had undergone STN-DBS (Devos et al., 2007; Moreau et al., 2012), but no improvements were observed in patients with moderate gait impairment without DBS (Espay et al., 2011). These differences could in part reflect differential pathologies in heterogeneous patient groups or selective medication effects. Future studies assessing noradrenergic stimulation could be complimented by specific imaging techniques that could relate any changes in neurotransmitter signal to clinical efficacy or lack thereof, such as 11C-MeNER PET, a highly selective noradrenaline transporter radioligand, and/or neuromelanin imaging, to assess the integrity of the locus coeruleus (Sommerauer et al., 2018).

Drugs already established in improving anxiety and depression (Takahashi et al., 2019) may also have beneficial effects on FOG. Both selective serotonin reuptake inhibitor (SSRI) and serotonin noradrenaline reuptake inhibitor (SNRI) treatment improved the FOG-Q after 10 weeks in a small group of Japanese PD patients with depression (Takahashi et al., 2019). Short-term administration of paroxetine (an SSRI) interestingly improved baseline walking speed in a small group of PD patients who were not premorbidly depressed, but did not augment the motor response to levodopa (Chung et al., 2005). Whilst anxiety and depression have been associated with FOG, it is not clear whether any symptomatic benefits of these agents may extend beyond their effects on mood. Similarly, cannabidiol (CBD) is also known to modulate brain areas involved with mood (Fusar-Poli et al., 2010; de Faria et al., 2020) and some work has reported reduced falls, pain, depression, and tremor in PD (Balash et al., 2017). The endocannabinoid system is linked to motor control and dopaminergic signalling, with the highest densities of cannabinoid type 1 (CB1) receptors located in the globus pallidus and substantia nigra (Babayeva et al., 2016). A double-blind phase II randomised controlled trial is ongoing to assess the efficacy of cannabidiol (CBD) on motor symptoms (UPDRS part III score) in 75 PD patients (ClinicalTrials.gov, 2021b). Whether these novel non-dopaminergic targets will benefit FOG will need further study.

## Surgical Approaches

Deep brain stimulation (DBS) provides access to deep brain structures and the ability to directly modulate networks implicated in the pathogenesis of FOG (Fasano et al., 2012). Conventional bilateral DBS of the subthalamic nucleus (STN-DBS) is generally considered to reduce Off-state FOG (Fasano et al., 2012; Vercruysse et al., 2014; Schlenstedt et al., 2017; Barbe et al., 2020) in addition to its robust effects on other motor symptoms (Fasano et al., 2012). STN-DBS appears to be effective for at least 3–5 years post implantation (Schlenstedt et al., 2017), but after this time it has been recognised that there is often worsening of gait and balance (Moro et al., 2010; Schlenstedt et al., 2017). A small proportion of patients who typically have longer disease duration (Barbe et al., 2020), less pre-operative dopamine responsiveness (Schlenstedt et al., 2017) and greater putamen grey matter atrophy (Karachi et al., 2019) have also been identified as experiencing increased FOG and falls shortly after STN-DBS and careful pre-operative screening is recommended (Karachi et al., 2019). Lowering the STN-DBS frequency to 60–80 Hz from the more conventional >100 Hz has been another approach that has been pursued with mixed success (Moreau et al., 2008). Meta-analysis data suggests low frequency stimulation induces greater reduction in observed FOG and FOG-Q scores compared to high frequency stimulation (Su et al., 2018), possibly relating to differential effects of stimulation frequency on pathological alpha and beta-band oscillations (Blumenfeld et al., 2015). These benefits are, however, commonly lost over a few weeks (Ricchi et al., 2012; Zibetti et al., 2016). Gait improvements with low frequency STN-DBS stimulation may also come at the cost of reduced tremor control in the off-medication state (Phibbs et al., 2014; Conway et al., 2021) though arguably this limitation is less of a concern in most patients who will continue to be titrated on levodopa.

Alternative stimulation strategies targeting non-STN structures, such as the pedunculopontine (PPN) area (Thevathasan et al., 2011) and the substantia nigra pars reticulata (SNr; Weiss et al., 2013) have also been investigated as potentially offering benefits to specifically improve FOG. The PPN is thought to play an important role in automatic gait through the release of pre-prepared movement (Garcia-Rill et al., 2019), whilst the SNr influences the PPN through efferent monosynaptic GABAergic transmission (Nandi et al., 2008). Typically, stimulation of the SNr has been interleaved with STN-DBS and studies with relatively small patient numbers have reported some alleviation of resistant gait impairment in PD (Weiss et al., 2013; Valldeoriola et al., 2019; Golfre Andreasi et al., 2020). Exactly where and how to best stimulate the PPN remains unclear with meta-analyses (Golestanirad et al., 2016; Wang et al., 2017; Yu et al., 2020) and collaborative efforts between expert centres revealing significant heterogeneity in the studies conducted to date (Hamani et al., 2016; Garcia-Rill et al., 2019).

It is well recognised that the traditional “open loop” DBS approach for PD requires external input to adjust stimulation parameters with the stimulation being delivered continuously without regard for fluctuating clinical or electrophysiological states. In contrast, “closed loop” DBS is now being explored with bidirectional devices that can both sense neural signals and deliver stimulation in response to specific electrophysiological changes, thus acting in real time. Such neural signals include prolonged beta (13–30 Hz) bursts (Anidi et al., 2018), and low beta (15–21 Hz) and theta (5–8 Hz) band oscillations (Chen et al., 2019) in the STN associated with FOG episodes, which have now been shown to attenuate with stimulation, strengthening their place as biomarkers for gait freezing. Recent work utilising this technological advance has shown that this approach may be feasible, demonstrating that in a single patient, closed-loop bilateral STN-DBS responding to STN beta band power was superior to conventional open-loop DBS in reducing the percentage of time spent freezing during a Stepping in Place task (Petrucci et al., 2020). Furthermore, work using a validated Virtual Reality gait paradigm in patients during STN-DBS lead implantation has identified an increase in pathological beta and theta rhythms just prior to freezing episodes that could provide a specific trigger signal for adjusting closed-loop systems on demand (Georgiades et al., 2019). Closed-loop work incorporating PPN-DBS have also begun but appear more problematic. One recent study implanted five medication-refractory FOG PD patients with two closed-loop PPN leads in addition to bilateral globus pallidus interna (GPi) leads (Molina et al., 2021). However, due to surgical complications, two of these patients needed explantation of the leads. Results from the remaining subjects were heterogeneous and may have been impacted by GPi co-stimulation.

These findings suggest that whilst DBS for FOG does offer potential, more studies with homogenous patient populations undergoing standardised procedures and assessments will be required to progress the field. In addition, it is likely that patients will need close monitoring over extended periods of careful treatment titration to optimise their clinical benefits (Bronte-Stewart et al., 2020).

## Non-Pharmacological Approaches

### Physical Rehabilitation

Whilst a number of guidelines for physiotherapy in PD exist (e.g., Keus et al., 2014), there is little specific guidance for addressing FOG. Physical rehabilitation is acknowledged to be crucial (Cosentino et al., 2020) and there are a number of approaches that have been applied to FOG in the research setting. These include action observation training (Pelosin et al., 2010, 2018; Agosta et al., 2017; Mezzarobba et al., 2020), treadmill training (Hong and Earhart, 2008; Frazzitta et al., 2009; Lo et al., 2010; Barbe et al., 2013; Picelli et al., 2016; Baizabal-Carvallo et al., 2020; Bekkers et al., 2020; Seuthe et al., 2020), aquatic obstacle training (Zhu et al., 2018), curved walking training (Cheng et al., 2017), supervised slackline training (Santos et al., 2017), as well as home based exercises (Canning et al., 2015). In contrast, general exercises and standard physiotherapy do not seem to be effective for the treatment of FOG (Miller et al., 2020). Behavioural strategies, such as cueing (Nieuwboer et al., 2007; Fietzek et al., 2014; Ginis et al., 2018), have also been extensively applied, as have dual-task situations (Geroin et al., 2018), which are designed to increase the complexity and recognise the association of FOG and selective cognitive deficits in attention (Naismith et al., 2010). Various types of cues (auditory, visual, somatosensory) and delivery systems (e.g., self-cueing, augmented reality) have been shown to positively modulate FOG (Fischer et al., 2018; Braunlich et al., 2019; Chang et al., 2019), though again the optimal way to target FOG is yet to be determined (Nieuwboer et al., 2007; Donovan et al., 2011; Spaulding et al., 2013; Young et al., 2016; Fischer et al., 2018; Braunlich et al., 2019; Chang et al., 2019). One meta-analysis comparing auditory to visual cues found that auditory cues appeared more effective, improving speed-related gait parameters in PD patients such as cadence and velocity as well as increasing step length whilst visual cues only improved step length (Spaulding et al., 2013). Auditory cues appear to make use of almost instantaneous motor entrainment to an external beat, activating the frontoparietal control and motor-cerebellar networks to bypass internal rhythm deficits of the basal ganglia (Braunlich et al., 2019). Somatosensory stimulation has historically been limited by the sophistication of the delivery technology, however, smaller wearable vibrotactile devices are emerging with early positive benefits on FOG (Tan et al., 2021), though their effects require validation. Long-term effects and the out-of-laboratory benefits of cueing training are to be confirmed (Chang et al., 2019). Methods to reduce cue habituation, including on-demand cueing, require further development before they can be deployed routinely (Ginis et al., 2018).

A recent meta-analysis of 19 studies involving 913 patients showed that interventions tended to have similar duration of each session (45–60 min) and number of sessions per week. Prolonged home based interventions (median 4 months) showed more promise of efficacy, whilst in terms of intervention categories, action observation and treadmill training had the most significant effect sizes (Cosentino et al., 2020). Common to these studies is the difficulty of creating a suitable control condition given the issues in blinding or finding a matched activity (e.g., cueing). In addition, only a limited number of studies have sought to correlate improvements in intervention with neurobiological changes through approaches such as fMRI (Silva-Batista et al., 2020). This can provide useful insights, such as a recent study that found increased activation in the mesencephalic locomotor region (MLR) post training in the intervention group of individual strength training with instability, but not in the control group of traditional strength training alone (Silva-Batista et al., 2020). The authors of this study also reported that these changes in MLR activation correlated with improvements in the NFOG-Q. It is likely that high-complexity exercises involving a combination of visual, cognitive, balance, and strength training have greater potential to modulate the network underlying FOG (Cosentino et al., 2020). Further larger trials investigating the long-term effects of therapy, the differences between On and Off-state training, and the comparison of multiple active intervention arms are desperately needed. Given that group training achieves similar positive effects to individual training (Pelosin et al., 2018), it is possible that such approaches could allow such programs to be delivered at scale.

### Neuropsychiatric Approaches

Cognitive and affective deficits certainly modulate FOG (Heremans et al., 2013; Shine et al., 2013c,d; Ehgoetz Martens et al., 2014; Walton et al., 2014; Muralidharan et al., 2016; Witt et al., 2019) and it should be appreciated that approaches like cognitive training, cognitive behavioural therapy and meditation all have the potential to improve FOG and a wider range of symptoms with no risk of harm. A small number of studies have been completed in this space and offer insights into future approaches. One recent randomised double-blinded study of 38 PD patients with FOG evaluated cognitive training specifically targeting those neuropsychological processes most strongly associated with the symptom, including inhibitory control, attentional set-shifting, working memory, processing speed and visuospatial skills (Walton et al., 2018). This intervention was provided over 12 weeks and resulted in a statistically significant reduction in actual FOG severity in patients during their On-state (Walton et al., 2018). A smaller randomised cross-over trial of 15 patients comparing cognitive training, cognitive behavioural therapy (CBT) and proprioceptive training replicated the positive effect of cognitive training on observed FOG severity but not NFOG-Q scores (Chow et al., 2021). Of interest, the anxiety-targeting CBT intervention exacerbated FOG whilst showing a trend toward improving the Parkinson Anxiety Scale (PAS; Chow et al., 2021). Proprioception training appeared to have the greatest effect, though it should be noted that the effects of each intervention were lost at 2 weeks after the 4-week training program (Chow et al., 2021).

Less standardised interventions are yet to be investigated for FOG. However, it has been reported that meditation may protect against grey matter atrophy (Last et al., 2017) and is already well accepted by PD patients with high perceived efficacy for alleviating affective and motor symptoms (Fitzpatrick et al., 2010; Donley et al., 2019). Though there are no trials examining the impact of mindfulness meditation on FOG, it does improve attention (Malinowski et al., 2017) and emotional regulation (Tang et al., 2015), which have both been recognised as important modulators of freezing. A recent randomised controlled trial in 138 PD patients found a yoga-mindfulness program significantly improved anxiety and depression scores over a stretching and resistance training control, in addition to their Unified Parkinson’s Disease Rating Scale Part III (motor) score (Kwok et al., 2019). A smaller study of just 30 PD patients participating in a yoga-meditation intervention experienced marked improvements in their FOG-Q, whereas a control group of no intervention did not change their freezing scores (Van Puymbroeck et al., 2018). It is unclear if these benefits were related to the meditation or physical rehabilitation component of the intervention (Van Puymbroeck et al., 2018).

Larger trials specifically investigating neuropsychiatric intervention strategies for FOG are now needed. These studies could potentially target both those with established FOG and an enriched population of at-risk patients. These studies will need to have much larger numbers than those already conducted, which will probably necessitate coordinated international multi-centre approaches where cross-over designs with multiple active arms may be the most efficient method to compare different techniques. These would ideally be conducted in combination with standardised objective measures of FOG and mechanisms for interpreting neurobiological changes such as functional neuroimaging [e.g., MRI (Silva-Batista et al., 2020)] or neurophysiological [e.g., EEG (Malinowski et al., 2017)] parameters.

### Non-invasive Brain Stimulation

Methods to modulate neuronal activity non-invasively also represent an attractive approach to access the distributed cortical and subcortical areas involved in FOG. Repetitive transcranial magnetic stimulation (rTMS), transcranial direct current stimulation (tDCS) and more recently, non-invasive vagal nerve stimulation (nVNS), have all been explored as potential options.

Non-invasive brain stimulation is thought to improve motor symptoms of PD by inducing focal release of endogenous striatal dopamine following stimulation of the ipsilateral cortex (Strafella et al., 2001, 2003), as well as increasing cortical excitability of motor and cognitive cortical areas involved in the upstream regulation of gait. Though there have been several sham-controlled studies investigating rTMS and tDCS for FOG (El-Tamawy et al., 2013; Lee et al., 2014; Valentino et al., 2014; Kim et al., 2015; Chang et al., 2017; Dagan et al., 2017; Lu et al., 2018; Ma et al., 2019; Mi et al., 2019), the optimal target, stimulation intensity and duration of treatment are yet to be confirmed. To illustrate the heterogeneity of the literature, though the majority of rTMS studies used high-frequency stimulation (≥10 Hz), the number of pulses and sessions varied significantly (450–3,000 pulses, delivered over 1–24 sessions) as well as treatment duration (3 days to 3 months) (Kim Y.W. et al., 2019; Xie et al., 2020). Indeed, two separate meta-analyses examining the benefits of prefrontal or primary motor cortical (M1) rTMS on FOG arrived at conflicting conclusions, though both noted heterogeneity amongst the included trials that may have masked a more positive outcome (Kim Y.W. et al., 2019; Xie et al., 2020). However, these studies do add to our understanding of brain networks involved in freezing. One resting-state functional MRI study with 10 sessions of rTMS delivered over an alternate target, the supplementary motor area (SMA), reported significant improvements in clinical freezing on the FOG-Q, as well as normalising functional connectivity patterns associated with FOG (Mi et al., 2020). Stimulation of a key cortical modulator confers effects on remote subcortical regions and demonstrates the related neural network with FOG (Mi et al., 2020). Previously, the SMA had not been thought to have a modulatory role on FOG based on single-session stimulation studies (Lee et al., 2014; Lu et al., 2018), suggesting that repeated sessions may be necessary to amplify the benefits of this type of intervention. Stimulation effects on FOG are likely transient rather than long-term, with a subgroup meta-analysis (Xie et al., 2020) of four rTMS studies with follow-up at ≥4 weeks (El-Tamawy et al., 2013; Ma et al., 2019; Mi et al., 2019, 2020) showing no significant difference in outcome by this time point. The effects of non-invasive stimulation may also be additive, as there appears to be a potential beneficial effect from multi-target compared to single target stimulation (Chang et al., 2017; Dagan et al., 2018; Manor et al., 2021). For example, simultaneous tDCS to the M1 and the left dorsolateral prefrontal cortex (DLPFC) improved freezing parameters immediately after the combined session, but not following primary motor cortex stimulation alone (Dagan et al., 2018). There is no additional benefit of simultaneous rTMS and rDCS stimulation compared to rTMS alone (Chang et al., 2017). Limitations of non-invasive stimulation are largely related to the need to remain within certain energy and pulse settings for safety, which reduces its access to deeper brain structures, but also contraindicates its use in PD patients with concomitant DBS (Magsood et al., 2020). However, taken altogether, these findings give cause for cautious excitement regarding the ability to modulate pathophysiological networks in FOG as techniques are further refined.

Vagus nerve stimulation (VNS) is an approved treatment for refractory epilepsy and depression that is also being investigated as a novel treatment for FOG in PD, especially following the availability of non-invasive transcutaneous stimulators (nVNS) (Farrand et al., 2017; Morris et al., 2019). It has been suggested that VNS may indirectly activate noradrenergic projections from the *locus coeruleus*, a region implicated in the pathogenesis of FOG, as well as exerting anti-inflammatory properties that may be important in halting disease progression. Recently, the first randomised, double-blind trial to investigate nVNS administered stimulation to the cervical vagus for 12 min each for 4 weeks in 33 PD patients with FOG (Mondal et al., 2021). The authors reported positive effects on gait velocity and step length, as well as reduced duration of freezing episodes in the laboratory gait assessment circuit, though interestingly patients’ perception of their FOG-related disability (FOG-Q score) did not improve (Mondal et al., 2021). Excitingly there was a significant reduction in biomarkers of inflammation [TNF-α, reduced-glutathione, and brain-derived neurotrophic factor (BDNF)] which may have implications for future disease modification trials.

Spinal cord stimulation (SCS) for FOG is also under investigation targeting spinal afferents to modulate cortical motor circuits (Reis Menezes et al., 2020). Despite several publications arising over the past decade using percutaneously inserted epidural spinal stimulators (Thevathasan et al., 2010; Agari and Date, 2012; Pinto de Souza et al., 2017; de Lima-Pardini et al., 2018; Fonoff et al., 2019; Hubsch et al., 2019), this approach has yet to find its place in routine clinical practice. This may relate to difficulty delivering long pulse width and high-frequency stimulation to reach deep spinal tissue, which drains battery life and increases unpleasant sensations in the patient, as well as limited scope for a sham device (Fonoff et al., 2019). More recently, the first non-invasive SCS study was published exploring transcutaneous magnetic stimulation to the fifth thoracic vertebra level in five PD patients (three sessions of 400 pulses at 5 Hz) (Reis Menezes et al., 2020). The authors reported significant improvements in NFOG-Q and UPDRS-III motor scores at 7 days following stimulation (Reis Menezes et al., 2020). Larger, sham-controlled studies are needed to establish if there is true benefit.

## What Approaches Could Help Us Identify a New Treatment?

To date, methods in randomised controlled trials to improve FOG are heterogeneous in timing, duration, type of intervention (single target vs. multitarget), and outcome measures. Most studies aim to improve FOG symptoms once they have developed, which may be too late in the disease process. There are, as yet, no studies using population enrichment strategies (age, biomarker characterisation, motor phenotype) to examine interventions in participants at high risk of developing FOG. Designing future trials in FOG might also require matching the candidate intervention to the subpopulation most likely to benefit. For example, a trial testing cognitive behavioural therapy might require a cohort of anxious freezers (Ehgoetz Martens et al., 2018b).

To inform such trials, exploratory studies to clarify the neurobiological components of freezing (e.g., imaging, neurophysiology, epidemiology) and to identify the most accurate ways to gather this data will be important. Objective non-gait freezing paradigms that quantify freezing frequency and duration such as Virtual Reality (VR) gait (Shine et al., 2013a), Stepping in Place (Nantel et al., 2011) and alternate finger tapping (D’Cruz et al., 2020; Trager et al., 2020) or handwriting (Heremans et al., 2019) for upper limb freezing correlate well with observed freezing behaviour and can also be combined with functional neuroimaging (Shine et al., 2013b). Studies to compare such models side-by-side to determine their sensitivity in distinct subgroups could then be used to inform the design of larger trials. Objective biomarkers for FOG, such as electrophysiological changes in beta-band power (Handojoseno et al., 2015; Marquez et al., 2020; Molina et al., 2020), could also be used to inform larger trials. Indeed, whilst DBS provides a unique opportunity to record continuously from deep brain structures, this would potentially interfere with other measurement modalities including MRI and EEG. Other dynamic imaging techniques, such as functional near infra-red spectroscopy (fNIRS; Maidan et al., 2015; Vitorio et al., 2020) or magnetoencephalography (MEG; Boto et al., 2018), need to be explored for use in FOG and may provide helpful insights into the phenomenon.

Wearable technology or home-based “smart” systems to non-invasively measure FOG in the community should become a priority. This would allow for long-term recording, providing the large number of training events needed for algorithms to learn freezing signals in the individual patient in order to subsequently predict FOG in real time. Deep learning has already been deployed to automatically detect gait freezing in video recorded walks (Hu et al., 2020) and also using real-time inertial measurements from wearable devices (Bikias et al., 2021). One group has recently developed an algorithm for use in patients without any previous anomalous gait data, trained on reference accelerometer data from a small group of reference normal and anomalous gaits, identifying 87.4% of FOG onsets (Bikias et al., 2021). Multi-modal measurements combining accelerometer and EEG readings are more accurate than single-modality measurement in detecting FOG events (Wang et al., 2020), suggesting future systems may require integration of different inputs. To create a multi-modal wearable system that is also comfortable to wear, it is likely that only the most robust signals from each modality will be included. Some progress has been made in identifying specific gait parameters that are the best for recognising abnormal steps (O’Day et al., 2020), and also in minimising intrusiveness of such devices, for example, the use of pressure-sensing insoles that were able to detect FOG in high agreement with clinical ratings (Pardoel et al., 2020).

There are also opportunities to make better use of already collected data. In a cross-sectional study of 172 PD patients, longer duration of treatment with dopamine agonists trended toward increased FOG, whilst longer duration of amantadine use trended against FOG, though these results did not reach significance in multiple regression (Giladi et al., 2001b). Collaboration between PD research groups to pool such data could prove useful. Interrogation of patient-level data in completed drug trials for potential candidate drugs for repurposing (e.g., if there was incidental reduction in fall frequency) could also provide a shortlist of already approved medications that can be investigated more cost-effectively. Efforts to follow large cohorts of PD patients prospectively with standardised biochemical, genetic and clinical assessments, such as in the Parkinson’s Progression Markers Initiative 2.0 (NCT04477785), are already underway. The addition of FOG-specific gait assessments to this dedicated study would greatly add to our understanding of how FOG develops and progresses, as well as allowing for an examination of triggering or protective factors.

## Conclusion

**FIGURE 2:**
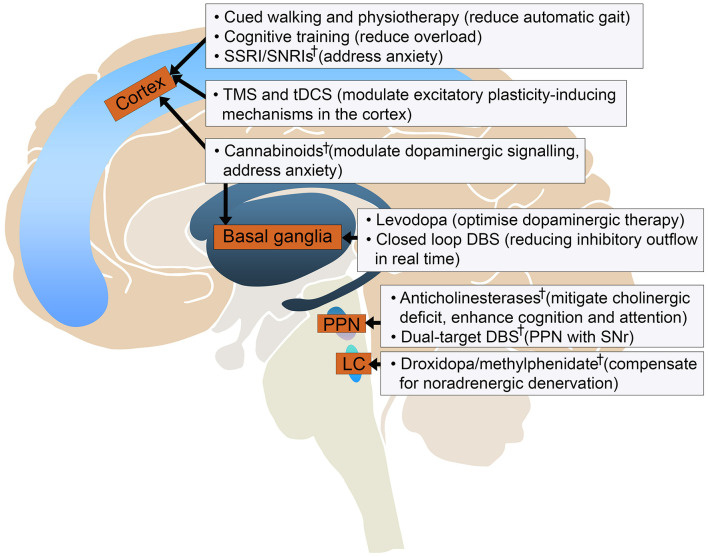
Current and experimental^†^ developments in treating gait freezing based on their potential targets in the locomotor circuit. DBS, deep brain stimulation; LC, locus coeruleus; PPN, pedunculopontine nucleus; SNr, substantia nigra pars reticulata; SSRI/SNRI, selective serotonin reuptake inhibitor/serotonin noradrenaline reuptake inhibitor; tDCS, transcranial direct current stimulation; TMS, transcranial magnetic stimulation.

This review summarises the major difficulties in understanding and treating FOG. What is apparent is that a multimodal approach will be crucial to tackle this problem (Figure 2). Collaboration between research centres to standardise FOG measurement and share patient datasets will be necessary to scale studies, in tandem with development of novel techniques to better understand its pathophysiology.

## Author Contributions

CC performed the literature search and manuscript writing. SL was responsible for project conception, structuring, and editing. Both authors contributed to the article and approved the submitted version.

## Conflict of Interest

The authors declare that the research was conducted in the absence of any commercial or financial relationships that could be construed as a potential conflict of interest.

## Publisher’s Note

All claims expressed in this article are solely those of the authors and do not necessarily represent those of their affiliated organizations, or those of the publisher, the editors and the reviewers. Any product that may be evaluated in this article, or claim that may be made by its manufacturer, is not guaranteed or endorsed by the publisher.
